# Digital therapeutics lead to clinically significant body weight loss in patients with metabolic dysfunction–associated steatotic liver disease: A systematic review and meta-analysis

**DOI:** 10.1097/HC9.0000000000000499

**Published:** 2024-07-31

**Authors:** Somaya Albhaisi, Justin Tondt, John Cyrus, Vernon M. Chinchilli, David E. Conroy, Jonathan G. Stine

**Affiliations:** 1 Department of Internal Medicine, Virginia Commonwealth University, Richmond, Virginia, USA; 2 Department of Family and Community Medicine, Penn State Health-Milton S. Hershey Medical Center, Hershey, Pennsylvania, USA; 3 Research & Education Department, Health Sciences Library, Virginia Commonwealth University, Richmond, Virginia, USA; 4 Department of Public Health Sciences, Penn State College of Medicine, Hershey, Pennsylvania, USA; 5 Department of Kinesiology, The Pennsylvania State University, University Park, Pennsylvania, USA; 6 Division of Gastroenterology and Hepatology, Department of Medicine, Penn State Health-Milton S. Hershey Medical Center, Hershey, Pennsylvania, USA; 7 Division of Gastroenterology and Hepatology, Fatty Liver Program, Penn State Health-Milton S. Hershey Medical Center, Hershey, Pennsylvania, USA; 8 Liver Center, Penn State Health-Milton S. Hershey Medical Center, Hershey, Pennsylvania, USA; 9 Cancer Institute, Penn State Health-Milton S. Hershey Medical Center, Hershey, Pennsylvania, USA

## Abstract

**Background:**

Most patients with metabolic dysfunction–associated steatotic liver disease are unable to achieve clinically significant body weight loss with traditional in-person approaches. Digital therapeutic (DTx)-delivered interventions offer promise to remove barriers to weight loss success inherent to traditional resource-heavy in-person programs and at a population level, but their efficacy remains relatively unknown.

**Methods:**

Published studies were identified through May 2023 by searching the following electronic databases: PubMed and Embase (Ovid). DTx intervention was compared to standard of care. The primary outcome was a change in body weight. Secondary outcomes included clinically significant body weight loss (≥5%) and change in liver enzymes.

**Results:**

Eight studies comprising 1001 patients met inclusion criteria (mean age: 47 y; body mass index: 33.2 kg/m^2^). The overall rate of clinically significant body weight loss was 33%, with DTx lifestyle interventions ranging from 4 to 24 months in length. DTx lifestyle intervention achieved statistically significant body weight loss (absolute change −3.4 kg, 95% CI: −4.8 to −2.0 kg, *p* < 0.01, relative change −3.9%, 95% CI: −6.6 to −1.3, *p* < 0.01) as well as clinically significant body weight loss of ≥5% (risk ratio: 3.0, 95% CI: 1.7–5.5, *p* < 0.01) compared to standard of care. This was seen alongside improvement in liver enzymes.

**Conclusions:**

DTx-delivered lifestyle intervention programs lead to greater amounts of body weight loss than traditional in-person lifestyle counseling. These results further support the role of DTx in delivering lifestyle intervention programs to patients with metabolic dysfunction–associated steatotic liver disease and suggest that this scalable intervention offers promise to benefit the billions of patients worldwide with this condition.

## Graphical abstract

**Figure:**
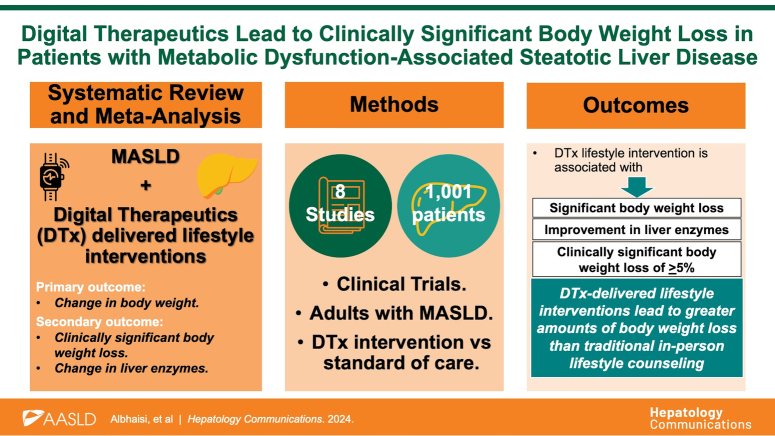

## INTRODUCTION

Metabolic dysfunction–associated steatotic liver disease (MASLD), formerly known as NAFLD, is defined as ≥5% liver fat and at least 1 of the 5 cardiometabolic criteria (Table 1).^1^ MASLD affects ~30% of the world population and encompasses both steatosis, which is the noninflammatory form, and metabolic dysfunction–associated steatohepatitis (MASH), which is the inflammatory form. If untreated, this common disease can progress to liver fibrosis, cirrhosis, and HCC.^2^ However, despite being one of the leading causes of liver disease, current treatment options are limited, although the recent FDA approval of resmetirom has provided the hepatology community with excitement as a pharmacologic treatment exists for those with stage 2–3 liver fibrosis.

**TABLE 1:** Cardiometabolic criteria for MASLD

1	(a) Body mass index ≥25 kg/m^2^ (23 kg/m^2^ in Asian populations) or (b) Waist circumference ≥94 cm for males, 80 cm for females, or ethnically adjusted value
2	(a) Fasting serum glucose ≥100 mg/dL or (b) 2-h post-load glucose ≥140 mg/dL or (c) Hemoglobin A1c ≥5.7% or (d) Antihyperglycemic drug use for type 2 diabetes
3	(a) Blood pressure ≥130/85 mm Hg or (b) Antihypertensive drug use
4	(a) Plasma triglycerides ≥150 mg/dL or (b) Lipid-lowering drug use
5	(a) Plasma HDL-cholesterol <40 mg/dL for males or 50 mg/dL for females or (b) Lipid-lowering drug use

Lifestyle intervention with a goal of 5% or more body weight loss is a cornerstone of MASLD clinical management.^3^ However, achieving and maintaining clinically significant weight loss is challenging, and many patients are unsuccessful. Self-reported barriers include lack of time, understanding, or access to lifestyle intervention resources.^4^ Moreover, weight stigma negatively impacts patients’ willingness to participate in weight loss interventions, and health care providers receive little formal training in weight loss interventions.^5^,^6^ As such, there is an unmet need to develop effective lifestyle interventions for patients with MASLD.

Recent advances in digital therapeutic (DTx) and mobile health (mHealth)-delivered interventions may reduce barriers to clinically significant weight loss typically associated with traditional in-person counseling. Examples of DTx-delivered and mHealth-delivered interventions include dietary intake trackers, fitness activity trackers, and real-time secure audio-visual technology.^7^ These have shown some success in the general population and small studies in patients with MASLD, but their impact on clinical outcomes in MASLD remains uncertain.^8^ Therefore, we conducted a systematic review and meta-analysis of clinical trials to determine if DTx-delivered and mHealth-delivered interventions in patients with MASLD lead to a clinically significant reduction in body weight.

## METHODS

We performed a systematic review of the existing medical literature in accordance with the Preferred Reporting Items for Systematic Reviews and Meta-Analyses (PRISMA, http://links.lww.com/HC9/A987) statement. This systematic review was registered with The International Prospective Register of Systematic Reviews (PROSPERO), an open-access online database of systematic review protocols (42023420308). Institutional review board approval was not required for this review.

### Identification of studies and searches

A detailed search was conducted by a Medical Librarian (John Cyrus) using indexing languages, including Medical Subject Headings and free text terms for NAFLD (search performed before nomenclature change), NASH, DTx, and lifestyle intervention. The search strategy can be found in Supplemental Table S1, http://links.lww.com/HC9/A988. We searched the following through May 2023: PubMed and Embase (Ovid). The gray literature (conference abstracts) was also searched through Embase. Following this, search results were imported into the Rayyan web and mobile app for systematic reviews (Qatar Computing Research Institute). To identify other potential studies to include, reference lists for all eligible studies were screened, as were identified systematic reviews and meta-analyses. Published abstracts were considered where appropriate.

### Study selection

Studies were chosen if they met the following inclusion criteria: (1) study design: clinical trials in human subjects; (2) population: adults (age ≥18 y) with MASLD and no other cause of liver disease, including secondary causes of hepatic steatosis; (3) exposure: DTx lifestyle intervention program; (4) outcome measures: provision of data to determine the primary or secondary outcome measures; and (5) English publication language. To fully extract the required data, we excluded abstracts where study investigators were unable to provide additional information as needed. All authors of this paper had access to the data, reviewed, and approved the final manuscript. The primary outcome was a change in body weight, both absolute and relative. Secondary outcomes were the rate of clinically significant body weight loss (≥5% or greater).^9^,^10^

### Data extraction and risk of bias assessment

Study-level data were extracted from each individual study, including author, country, study conditions (DTx vs. control), and study year. Subject-level data were also extracted and included age, sex, body mass index, and the primary and secondary outcomes. To complete data extraction, authors were contacted for unpublished data. Three author groups contributed unpublished data pertinent to the clinically significant body weight loss secondary outcome. Double coding was performed to verify the data extracted.

To adjudicate individual study risk of bias, either the Cochrane Risk of Bias Tool Version 2 (ROB2) for randomized studies or ROBINS-I tool for nonrandomized studies were used. The ROB2 tool has 5 domains: (1) randomization process; (2) deviations from intended intervention; (3) missing outcome data; (4) outcome measurement; and (5) selection of the reported result. For each domain, each individual study was graded as yes, partly yes, partly no, no, or no information. Following this assessment, each domain was then assigned a risk of bias: (1) low; (2) some concerns; and (3) high. The ROBINS-1 tool has 7 domains: (1) confounding; (2) selection of participants into the study; (3) classification of intervention; (4) deviations from intended interventions; (5) missing data; (6) measurement of outcomes; and (7) selection of the reported result. Following this assessment, each domain was then assigned an overall risk of bias: (1) low; (2) moderate concerns; (3) serious; and (4) critical. Each reviewer (Somaya Albhaisi, Justin Tondt, and Jonathan G. Stine) was trained in both the ROB2 and ROBINS-I tools with online training.

### Statistical analysis

To guide data analysis, the Cochrane Handbook for Systematic Reviews of Interventions was used. The number of subjects with a recorded body weight before and after DTx were extracted for each individual study. Both the absolute and relative change in body weight were extracted for each study, as was the corresponding SD. For clinically significant body weight loss, subjects were extracted into 2 groups, those who had a ≥5% reduction in body weight versus those who did not.

Review manager software (Rev-Man version 5.4; Copenhagen; The Nordic Cochrane Centre; The Cochrane Collaboration; September 2020) was used to perform both qualitative and quantitative analyses. Where appropriate, mean differences (absolute and relative body weight) were calculated, and pooled risk ratios between the 2 groups of subjects for each outcome were estimated by weighting the study-specific risk ratios by the inverse of their individual variance. To determine 95% CIs, DerSimonian and Laird random-effects models were used. Study variability (between) was assessed using the Cochran’s Q statistic (*p* < 0.05). The *I*^2^ index was calculated to quantify the proportion of heterogeneity accounted for by between-study variability. Given <10 studies were included in this review, formal statistical analysis for publication bias was not pursued.^11^ Random-effects meta-regression analysis^12^ was performed for 2 study-level regressors, study duration and study percentage of biological females. Given the significant heterogeneity in DTx intervention, intervention-specific regressors beyond intervention duration were unable to be analyzed with confidence. Sensitivity analysis was performed by limiting only studies to adults with MASH.

## RESULTS

### Study selection

**FIGURE 1:**
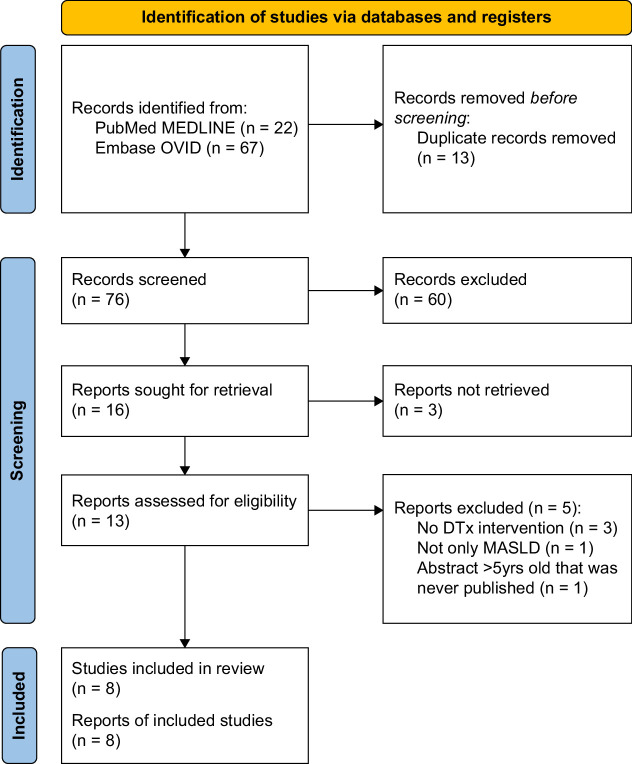
PRISMA diagram. Abbreviations: DTx, digital therapeutic; MASLD, metabolic dysfunction–associated steatotic liver disease. PRISMA, Preferred Reporting Items for Systematic Reviews and Meta-Analyses

The database search identified a total of 76 abstracts and titles after duplicate removal. After a review of all titles, abstracts, and full study texts, a total of 8 studies met the inclusion criteria (Figure 1).^13^,^14^,^15^,^16^,^17^,^18^,^19^,^20^ Supplemental Table S2, http://links.lww.com/HC9/A988, details the reasons for study exclusion following full-text publication review as well as study citations.

### Study and patient characteristics

Eight studies comprising 1001 patients met the inclusion criteria. Study and patient characteristics are shown in Table 2. Study intervention duration ranged from 4 to 24 months and included the following lifestyle interventions: 5 mHealth-delivered and 2 web-based. Of the mHealth platforms, 3 included smartphone applications, 1 of which is commercially available. Each intervention included counseling about lifestyle intervention as the cornerstone of the intervention. Three studies enrolled only adults with MASH. Table 2 summarizes additional characteristics of each DTx intervention. The subject’s mean average age was 47 years (range: 42–55 y); 42% of the participants were female and 36% with diabetes. The subject’s mean body mass index was 33.2 kg/m^2^ (range: 30.0–39.0 kg/m^2^).

**TABLE 2:** Characteristics of included studies

First author (Reference) and country	Study design	Study length	MASLD diagnosis	Subjects	Demographics	Intervention group	Control group	Outcomes
Axley et al, USA^13^	RCT	24 wk	Steatosis on ultrasound + exclusion of other liver disease, including self-reported alcohol use of ≥10 g/d (cirrhosis without hepatic decompensation included)	Overall: 30 Intervention: 13 enrolled, 13 analyzed Control: 17 enrolled, 17 analyzed Cirrhosis without hepatic decompensation: 14 (6 intervention, 8 control)	Age: Intervention: 54 years Control: 52 years BMI: Intervention: 39 kg/m^2^ Control 36 kg/m^2^ Female: Intervention 85% Control 47% Diabetes: Intervention 38% Control 30%	Text messaging intervention providing education (e.g., nutrition, exercise, stress management), motivation and actionable advice messages	Not described	Dropout rate: 39% intervention vs. 18% control Mean body weight change: −2.6% intervention vs. +0.1% control Mean AST (n = 30) = −9 IU/L intervention vs. ±0 IU/L control Mean ALT (n = 30) = −12 IU/L intervention vs. ±0 IU/L control
Lim et al, Singapore^14^	RCT	24 wk	AASLD guideline criteria for MASLD^21^	Overall: 108 Intervention: 55 enrolled, 55 analyzed Control: 53 enrolled, 53 analyzed	Age: Intervention: 47 y Control: 46 y BMI: Intervention: 30.1 kg/m^2^ Control 30.8 kg/m^2^ Female: Intervention 42% Control 32% Diabetes: Intervention 20% Control 36%	Single face-to-face dietician visits followed by remote support through the Nutritionist Buddy mobile application	Standard clinical care, including counseling on American Heart Association dietary and physical activity modifications by a trained nurse in a single face-to-face session	Dropout rate: 9% intervention vs. 4% control Mean body weight change: −4.4% intervention vs. −0.6% control Mean AST (n = 104) = −17 IU/L intervention vs. −7 IU/L control Mean ALT (n = 103) = −34 IU/L intervention vs. −12 IU/L control Mean systolic blood pressure (n = 72) = −12 mm Hg intervention vs. −2 mm Hg control
Mazzotti et al, Italy^15^	Open-label clinical trial	24 mo	Not specified	Overall: 716 In-person group-based intervention (GBI): 438 enrolled, 301 analyzed Web-based intervention (WBI): 278 enrolled, 118 analyzed	Age: GBI: 55 y WBI: 46 y BMI: GBI: 33.2 kg/m^2^ WBI: 33.7 kg/m^2^ Female: GBI: 33% WBI: 55% Diabetes: GBI: 41% WBI: 22%	GBI: 5 weekly sessions of 120 min chaired by a physician or psychologist in groups of 20–25 persons with counseling about the Mediterranean diet, physical activity, energy balance, weight monitoring, portion size, food shopping and labels, and behavioral strategies WBI: Reproduces the group program with 5 weekly sessions and includes interactive slides, tests, and gamification	None	24-mo dropout rate: 31% GBI vs. 58% WBI Mean body weight change: −4.0% GBI vs. −5.5% WBI Mean ALT = −19 IU/L GBI vs. −22 IU/L WBI Mean physical activity = +7.7 MET/h/wk GBI vs. +9.1 MET/h/wk
Motz et al, USA^16^	Adaptation of clinical trial to COVID-19 restrictions, Single-arm	20 wk	Biopsy-proven MASH according to NASH CRN criteria^22^	Overall: 3 Intervention: 3 enrolled, 3 analyzed	Age: 52 y BMI: 31.9 kg/m^2^ Female:100% Diabetes: NA	Moderate-intensity aerobic exercise 5 d a week under real-time supervision by an exercise physiologist using synchronous audio/video	None	All completed ≥80% of sessions Mean MRI-PDFF relative reduction = 35.1% Mean HbA1c = −0.5% Mean HOMA-IR = −4.0 Mean AST = −8.5 IU/L Mean ALT = −12.5 IU/L Mean VO_2_peak = +9.9 mL/kg/min
Pfirrmann et al, Germany^23^	Single-arm	12 wk	Histologically confirmed MASLD	Overall: 44 Intervention: 44 enrolled, 44 analyzed	Age: 42 y BMI: 31.9 kg/m^2^ Female: 32% Diabetes: NA	Web-based platform for individualized exercise training support with 3 exercise sessions per week (aerobic and resistance training) and regular weekly feedback and exercise progression	None	Dropout: 2% Mean body weight change: −1% Mean VO_2_peak = +2.4 mL/kg/min
Sato et al, Japan^18^	Single-arm	48 wk	Histologically confirmed MASH according to NASH CRN criteria	Overall: 20 Intervention: 20 enrolled, 19 analyzed	Age: 52 y BMI: 32.0 kg/m^2^ Female: 47% Diabetes: 11%	NASH App mobile application	None	67% app engagement at 48 wk Dropout NR Mean NAS change = −2.1 Mean body weight change = 7.3 kg Mean HbA1c = −0.3% Mean AST = −23 IU/L Mean ALT = −40 IU/L Mean HOMA-IR = −4.94
Stine et al, USA^19^	RCT	16 wks	Histologically confirmed MASH according to NASH CRN criteria or hepatic steatosis on imaging + noninvasive test suggesting MASH (eg, FIB-4 ≥1.45, VCTE liver stiffness >8.2, FAST score >0.35)	Overall: 40 Intervention: 20 enrolled, 20 analyzed Control: 20 enrolled, 20 analyzed	Intervention: 53 y Control: 50 y BMI: Intervention: 36.1 kg/m^2^ Control 36.3 kg/m^2^ Female: Intervention 60% Control 85% Diabetes: Intervention 50% Control 40%	Noom Weight mobile application in addition to standard clinical care. Bluetooth scale provided	Standard clinical care, including counseling from a hepatologist on the Mediterranean diet and 150 min/wk of moderate-intensity physical activity. Bluetooth scale provided	Dropout rate: 25% intervention vs. 10% control Mean body weight change: −5.4% intervention vs. −0.4% control No between-group difference in AST, ALT, fasting glucose, or platelet count (only 40% with reportable data)
Tincopa et al, USA^20^	Single-arm	24 wk	AASLD guideline criteria for MASLD, including adults with compensated cirrhosis	Overall: 40 Intervention: 40 enrolled, 40 analyzed Compensated cirrhosis: 11	Age: 53 y BMI: 33.9 kg/m^2^ Female: 47% Diabetes: 43%	Fitness activity tracker provided with weekly, progressive step goals	NA	Dropout: 18% Mean body weight change: ±0% Mean ALT change: −2.5 IU/L Mean A1c change: −0.1%

Abbreviations: ALT, alanine aminotransferase; AST, aspartate aminotransferase; BMI, body mass index; Hb, hemoglobin; HOMA-IR, homeostatic model assessment for insulin resistance; MET, Metabolic Equivalents of Task; PDFF, proton density fat fraction; RCT, randomized controlled trial; VO_2_peak, peak oxygen consumption.

### Primary outcome: Change in body weight

**FIGURE 2:**
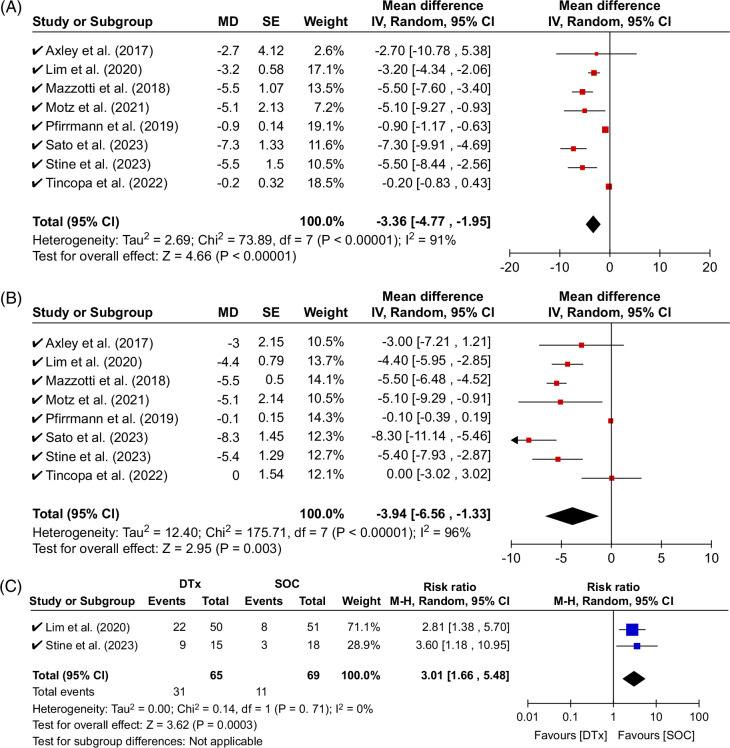
Pooled efficacy of DTx in leading to body weight loss in patients with MASLD. (A) Mean body weight loss with DTx is nearly 3.5 kg; (B) mean relative body weight loss with DTx is nearly 4%; (C) subjects achieve 5% body weight loss or greater 3-fold more often with DTx than SOC. Abbreviations: DTx, digital therapeutic; MASLD, metabolic dysfunction–associated steatotic liver disease; SOC, standard of care.

Eight studies contributed data to the analysis of the primary outcome with 1001 subjects. DTx lifestyle intervention achieved statistically significant body weight loss (absolute change −3.4 kg, 95% CI: −4.8 to −2.0 kg, *p* < 0.01, relative change −3.9%, 95% CI: −6.6 to −1.3, *p* < 0.01) (Figures 2A, B). High study heterogeneity was observed both for absolute (*I*^2^ = 91%) and relative change (*I*^2^ = 96%) in body weight.

### Secondary outcomes: Clinically significant body weight loss

The overall rate of clinically significant body weight loss was 33% (53 of 160 DTx patients with available data). Two studies with 134 subjects (65 DTx and 69 control conditions) were included in the secondary outcome of clinically significant body weight loss. Meta-analysis demonstrated DTx subjects achieved a clinically significant body weight loss of ≥5% (risk ratio: 3.01, 95% CI: 1.66–5.48, *p* < 0.01) more often than the standard of care (Figure 2C). No study heterogeneity was observed (*I*^2^ = 0%).

### Secondary outcomes: Change in liver enzymes

Informed by 8 studies, the overall reduction with DTx in alanine aminotransferase was −23.3 ± 6.3 IU/L and for 6 studies, aspartate aminotransferase −15.0 ± 3.5 IU/L. When compared to standard of care, DTx led to a mean difference in alanine aminotransferase of −11.5 IU/L (95% CI: −24.3 to 1.3 IU/L, *p* = 0.05) and a mean difference in aspartate aminotransferase of −5.8 IU/L (95% CI: −12.8 to 1.3 IU/L, *p* = 0.09) (Supplemental Figure S1, http://links.lww.com/HC9/A988).

### Sensitivity analysis

Given the relationship between body weight loss and histologic MASH improvement, we performed sensitivity analysis by limiting the analysis to include only studies enrolling adults with MASH.^16^,^18^,^19^ Across these 3 studies, the findings were even more robust. Overall weight loss was −6.3 kg (95% CI: −8.0 to −4.5, *p* < 0.001), and −6.4% weight loss was seen (95% CI: −8.5 to −4.4, *p* < 0.001). When limiting only to MASH studies, the heterogeneity was significantly reduced with *I*^2^ = 0% and 26% for each respective analysis (Supplemental Figure S2, http://links.lww.com/HC9/A988).

### Meta-regression analysis

We performed a random-effects meta-regression analysis [reference] with 2 study-level regressors, namely, study duration and study percentage of females, to determine their impact on the 2 outcome variables. Neither of the study-level regressors was statistically significant (*p* = 0.10 and 0.15 for study duration and study percentage of females, respectively, for the absolute outcome; *p* = 0.14 and 0.25 for study duration and study percentage of females, respectively, for the percent outcome).

### Risk of bias assessment

**FIGURE 3:**
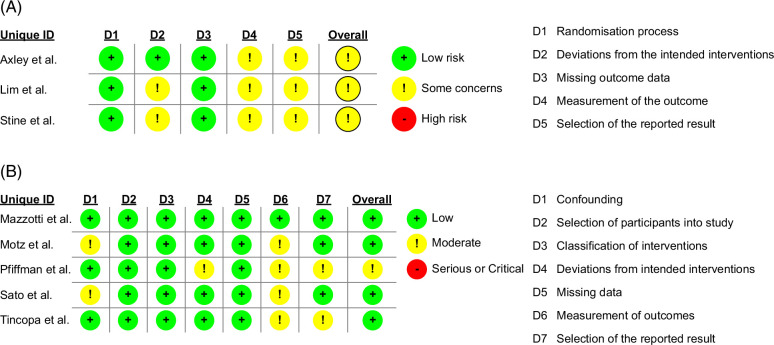
Risk of bias. (A) Risk of bias using ROB2; (B) risk of bias using ROBINS-I tool. Most included studies were adjudicated to have low bias.

Most studies were adjudicated to have a low or moderate risk of bias (Figure 3). No study had a high bias.

## DISCUSSION

This systematic review and meta-analysis found a consistent body of evidence from multiple international clinical trials demonstrating that DTx lifestyle intervention can achieve clinically significant body weight loss in patients with MASLD. DTx lifestyle intervention leads to a clinically meaningful reduction in body weight by a factor of just over 3 when compared to standard clinical care, which typically involves in-office counseling regarding Mediterranean-informed dietary change and increased physical activity. This weight loss was observed in parallel with a reduction in liver enzymes. To our knowledge, this is the first systematic review and meta-analysis evaluating the effectiveness of DTx-delivered lifestyle interventions in achieving long-term weight loss compared to traditional lifestyle interventions in patients with MASLD. Of high clinical relevance, the greatest weight loss was seen in adults with MASH, an important finding given the relationship between body weight loss and improvement in histologic MASH activity.

Emerging evidence supports that DTx lifestyle interventions can be effective in improving various aspects of health, including weight management, mental well-being, and chronic disease management. These interventions use mobile apps, wearable devices, and online platforms to deliver personalized interventions and support to individuals.^24^,^25^ By harnessing the power of technology, DTx have the potential to revolutionize health care by providing accessible and scalable solutions that can be tailored to individual needs and preferences.^26^ With further research and development, these interventions may become an integral part of preventive and therapeutic health care strategies, especially given our findings that DTx interventions lead to greater body weight loss than in-person interventions.^15^,^27^

Adopting DTx lifestyle interventions has been shown to have numerous benefits for individuals, including improved overall health and well-being.^28^ These interventions have been found to reduce the risk of chronic diseases such as diabetes and heart disease, increase energy levels, and enhance mental clarity. Individuals who follow DTx lifestyle interventions may also experience weight loss, improved sleep quality, and better stress management.^29^ These interventions typically involve nutrition plans, regular physical activity, and mental health support, which have been proven to bring about long-term sustainable changes that positively impact one’s quality of life.^30^

Despite our long-standing knowledge that traditional lifestyle interventions based on diet and exercise are successful, achieving moderate weight loss and durable lifestyle changes remain difficult obstacles for physicians and patients. Previous studies cited several factors as the causes of failure, including boredom of long-term weight reduction, particularly among patients with weak self-management abilities, and health care professionals’ inability to give real-time out-of-hospital supervision and psychological support.^31^ In addition, because traditional treatment approaches rely on in-person interactions, there are problematic elements, including inconvenient consultation times, exorbitant offline transportation costs, and challenging offline medical procedures, which are obviously demotivating to treatment adherence. Aside from those, standard lifestyle therapies have faced additional difficulties due to the quick emergence and widespread transmission of COVID-19. Previously adherent individuals experienced travel restrictions and were unable to access routine medical care during this time due to home isolation and limited mobility, which exacerbates the disease’s progression.^32^ The use of DTx in the treatment of MASLD is still in the exploratory stage, and there are currently few studies that specifically report DTx for MASLD. Some researchers have carried out remote lifestyle interventions using web-based platforms, including diet, exercise, and health education. This can be seen as a first step in the practical investigation of DTx for patients with MASLD. The differences in weight loss between DTx lifestyle interventions and traditional approaches may reflect that adherence is the most significant factor in achieving successful weight loss outcomes, especially for individuals with MASLD. DTx interventions have demonstrated higher adherence rates compared to traditional lifestyle interventions, including studies which were included in this meta-analysis, which documented adherence rates ranging from 42% to 100%.

### Strengths and limitations

This systematic review provides comprehensive evidence of the value of lifestyle interventions based on exercise, diet, or their combination in achieving significant weight loss and improving clinical outcomes in patients with MASLD. Our study has several strengths, which include the inclusion of only clinical trials with a diverse population of overweight and obese adults with MASLD. The included studies evaluated different DTx interventions. Meta-regression did not find any significant impact for either study length or the female sex, providing high confidence and scientific rigor when interpreting the study results. The finding of the most robust body weight loss in adults with MASH is of high clinical importance given the percentage of body weight loss approximated what is accepted to improve liver fibrosis, the intermediate endpoint most closely tied to future liver-related events and mortality.

On the other hand, there are several limitations of the current study which is based on the existing body of scientific evidence regarding DTx lifestyle intervention in individuals with MASLD. There was significant heterogeneity among the included studies, which may limit the generalizability of the study findings. The considerable heterogeneity may be attributable to both the population studied as all stages of MASLD (except decompensated cirrhosis) were included in this analysis as study-level data was not granular enough to phenotype participants into liver fibrosis stages and also the DTx interventions themselves, which varied in MASLD-specificity, use of wearable devices, educational content, and degree of personalization. Other limitations include no long-term follow-up beyond 1 year. We also cannot completely rule out the possibility of publication bias even though we exhaustively reviewed the literature for published studies given the potential that studies reporting no significant effects are often unpublished, and we did not perform a registry search. As opposed to patient-level data, we could only obtain aggregate data, which might have an impact on the effect estimates. In addition, most trials lacked information on the impact of weight loss on other metabolic health markers, so we were unable to draw definitive conclusions regarding whether DTx lifestyle interventions improved cardiovascular health. Moreover, not all data on the indicators of MASLD resolution were available across most included trials. Another limitation was the inclusion of only studies published in English. Lastly, due to the wide variation of implemented protocols in the included clinical trials, no conclusions could be made regarding the best modality of DTx interventions or optimal exercise or diet. This is an intriguing avenue ripe for future research and should be considered a priority by funding agencies. We would suggest the development of an ideal DTx lifestyle intervention should include the following elements: (1) specificity for adults with MASLD; (2) incorporation of wearable devices; (3) delivered through wireless networks; and (4) use input-relevant information captured from user input into the DTx platform combined with the wearable device to create a customized, personalized management plan. The personalized management plan should include screening, brief educational intervention, and treatment focusing on (1) dietary intake; (2) exercise training; (3) medication administration; (4) sleep quality; (5) psychology of human behavior and motivation; and (6) mindfulness.

## CONCLUSIONS

In conclusion, DTx-delivered lifestyle intervention programs lead to clinically significant body weight loss 3-fold more often than the standard of care. These results further support the role of DTx to deliver lifestyle intervention programs to patients with MASLD and suggest that this scalable intervention offers promise to benefit the billions of patients worldwide who are living with MASLD.

## Supplementary Material

SUPPLEMENTARY MATERIAL
