# Supplementary material for: Digital therapeutics lead to clinically significant body weight loss in patients with metabolic dysfunction–associated steatotic liver disease: A systematic review and meta-analysis
Source: Hepatol Commun. 2024 Jul 31;8(8):e0499. doi: 10.1097/HC9.0000000000000499 (PMC12333739; doi:10.1097/HC9.0000000000000499)
Supplement: SUPPLEMENTARY MATERIAL [file hc9-8-e0499-s002.docx]

**Supplementary Table 1. Search Strategy**

| Information | | |
| --- | --- | --- |
| Search date: April 14, 2022 | | |
| Databases searched: PubMed MEDLINE, Embase (Ovid) | | |
| Limits: n/a | | |
| # | Searches | Results |
| **PubMed MEDLINE** | | |
| 1 | (("non alcoholic fatty liver disease"[MeSH Terms] OR "non alcoholic fatty liver disease"[tiab] OR NAFLD[ot] OR NASH[ot] OR "Non Alcoholic SteatoHepatitis"[tiab]) |  |
| 2 | ("Life Style"[Mesh] OR "lifestyle"[tiab] OR "life style"[tiab] OR Diet[tiab] OR "healthy eating"[tiab] OR "diet"[Mesh] OR "diet therapy"[mesh] OR "Exercise"[Mesh] OR "Exercise Therapy"[Mesh] OR exercise[tiab] OR "physical activity"[tiab] OR "endurance training"[tiab] OR "strength training"[tiab] OR "weight loss"[mesh] OR "weight loss"[tiab] OR "weight reduction"[tiab])) |  |
| 3 | (telehealth OR "tele health" OR telemedicine OR "tele medicine" OR "mobile health"[Title/Abstract:~2] OR mhealth OR "m health" OR ehealth OR "e health" OR "digital health"[Title/Abstract:~2] OR "digital therapeutics"[Title/Abstract:~2] OR "mobile phone" OR smartphone OR wearable*[tiab] OR "remote monitor"[Title/Abstract:~2] OR "remote monitoring"[Title/Abstract:~2] OR "mobile app"[Title/Abstract:~2] OR "mobile application"[Title/Abstract:~2]) |  |
| 4 | 1 AND 2 AND 3 | 22 |
|  |  |  |
| # | Searches | Results |
| **Embase** |  |  |
| 1 | exp nonalcoholic fatty liver/ or NAFLD.ti,ab,kw. or NASH.ti,ab,kw. or "non alcoholic ajd3 fatty liver".ti,ab,kw. or "non alcoholic ajd3 steatohepatitis".ti,ab,kw. |  |
| 2 | exp lifestyle/ or exp diet/ or exp diet therapy/ or exp exercise/ or exp kinesiotherapy/ or exp body weight loss/ or ("lifestyle" or "life style" or diet or "healthy eating" or exercise or "physical activity" or "endurance training" or "strength training" or "weight loss" or "weight reduction").ti,ab,kw. |  |
| 3 | exp telehealth/ or (telehealth or "tele health" or telemedicine or "tele medicine" or (mobile adj2 health) or mhealth or "m health" or ehealth or "e health" or (digital adj2 health) or (digital adj2 therapeutics) or "mobile phone" or smartphone or wearable* or (remote adj2 monitor*) or (mobile adj2 app*)).ti,ab,kw. |  |
| 4 | 1 AND 2 AND 3 | 67 |
|  |  |  |

**Supplementary Table 2. Studies excluded after full-text review^1-5^**

1. Kwon OY, Kim SU, Ahn SH, et al. Self-Management and Associated Factors among Patients with Non-Alcoholic Fatty Liver Disease: A Cross-Sectional Study. Int J Environ Res Public Health 2022;20. **No digital intervention.**

2. Policarpo S, Machado MV, Cortez-Pinto H. Telemedicine as a tool for dietary intervention in NAFLD-HIV patients during the COVID-19 lockdown: A randomized controlled trial. Clin Nutr ESPEN 2021;43:329-334. **HIV co-infection.**

3. Ghodsbin F, Javanmardifard S, Javad Kaviani M, et al. Effect of tele-nursing in the improving of the ultrasound findings in patients with nonalcoholic fatty liver diseases: A Randomized Clinical Trial study. Invest Educ Enferm 2018;36. **No digital therapeutic intervention.**

4. Repking S, Mayer K, Folkers C, et al. An Evaluation of an Advanced Practice Provider Led Weight Intervention in Liver Disease (WILD) Pathway. Gastroenterol Nurs 2023. **No digital therapeutic intervention.**

5. Tozzi A DFM, Giangrandi I, Novara E, Sofi F, Casini A. NUNA NUTRITIONAL NAVIGATOR SMARTPHONE FREE APPLICATION FOR IMPROVING ADHERENCE TO MEDITERRANEAN

DIET AND REDUCING BODY WEIGHT IN NON-ALCOHOLIC FATTY LIVER DISEASE PATIENTS: A

PILOT STUDY. Digestive and Liver Disease 2016;OC.03.3. **Abstract >5 years old never published**

**in manuscript form.**

**Supplementary Figure S1- Change in liver enzymes comparing digital therapeutic lifestyle intervention to standard of care**

**
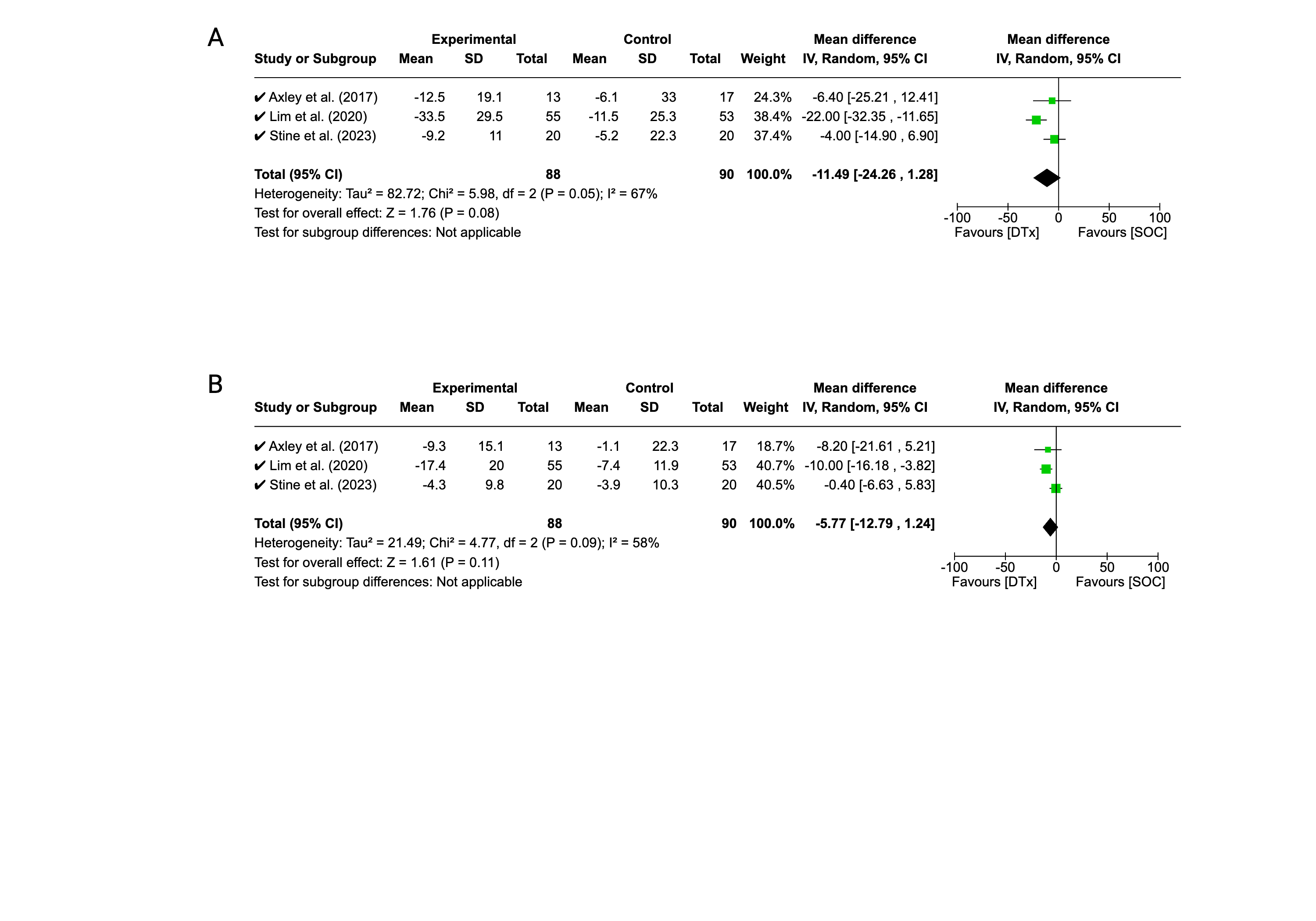
**

**(A) Change in ALT approached statistical significance when comparing DTx to SOC; (B) AST reduction, while ALT, also approached statistical significance**

**Supplementary Figure S2- Sensitivity analysis limiting only to studies enrolling adults with MASH**


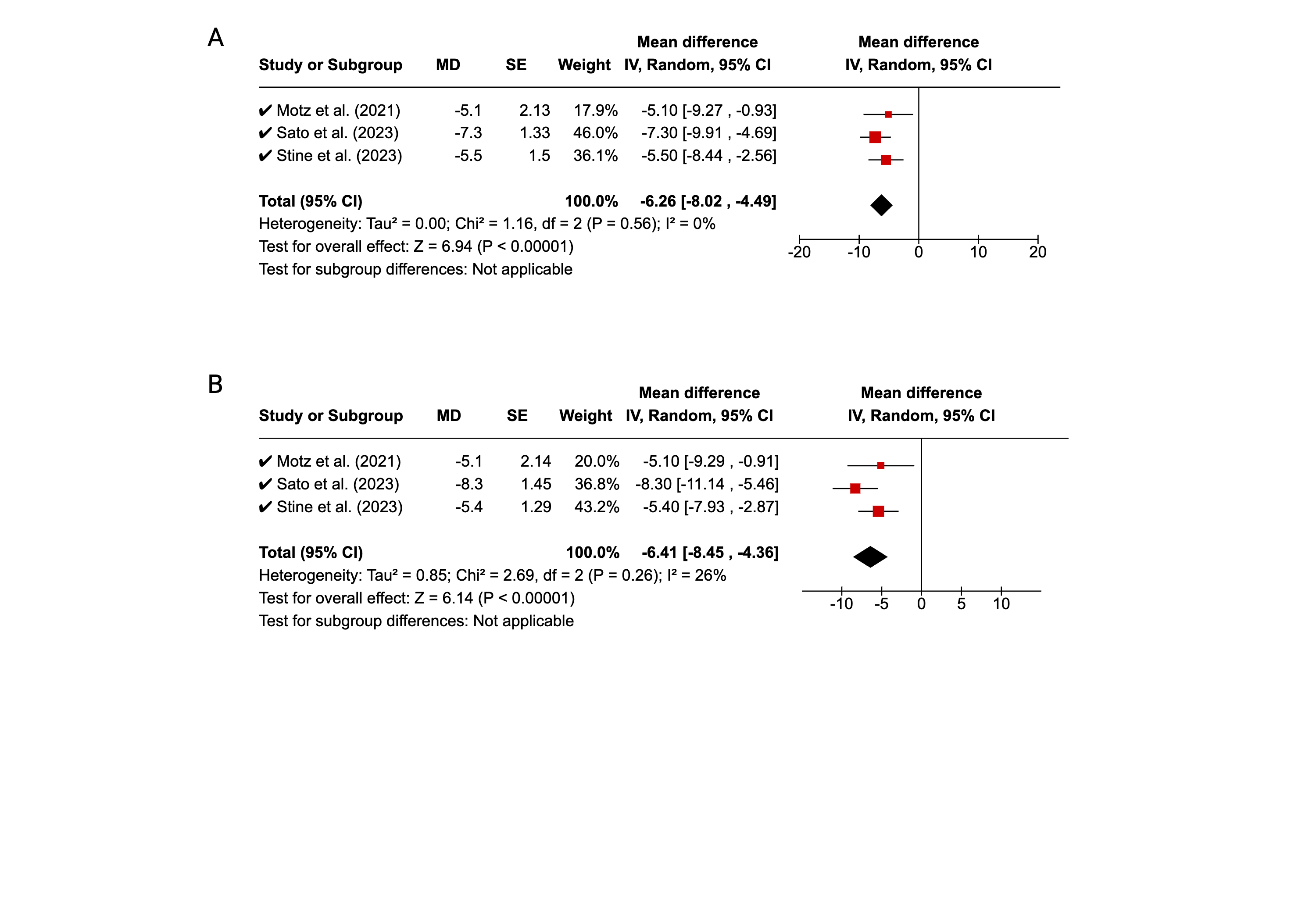


**(A) Mean body weight loss with DTx was -6.3kg; (B) Mean relative body weight loss was nearly 7%, the threshold of weight loss for MASH that is known to improve liver histology**
